# Complete Hospital Co-Operation

**Published:** 1906-06-30

**Authors:** 


### The Hospital

A Journal of

The Medical Sciences and Hospital Administration.

Vol. XL.—No. 1,032. SATURDAY, JUNE [illegible]0 1906.

### Complete Hospital Co-operation.

When the history of the hospital system in Great Britain comes to be fully written, large recognition will doubtless be paid to the movement by which in recent years the hospitals have been converted from independent and isolated units into members of a great organisation. The principle underlying this movement has, it is true, not yet been fully applied, but its recognition constituted a great step in advance, and the enormous economic advantages which have followed even its partial adoption cannot fail to attract the attention and interest of the thoughtful historian. The immediate future may be expected to bring a further application of business method to the co-ordination of the various agencies by which attempts are made to secure efficient medical advice and assistance for the poorer members of the community. The out-door departments of hospitals, provident and Poor-law dispensaries, and various nursing associations, are all engaged in activities of very similar order. Yet with but few exceptions there is no efficient arrangement to secure communication between them, and consequently there is much overlapping and much waste both of time and effort. It may not be easy to devise a system in which these various agencies may be made to play each its appropriate part. The task, however, is certainly not beyond the wit of man, and it is one which must be faced if efficiency and economy are to be secured. In his recent address to the Metropolitan Provident Medical Association Sir Wm. Church directed attention to this question and urged co-operation between hospitals, provident dispensaries, and nursing societies as the only way by which the abuse of charities could be prevented. Such abuse has, obviously, a special interest for members of the medical profession, but it concerns also all who recognise the necessity for the economic management of charitable funds as well as those who value the encouragement of thrift and personal independence in the life of the nation. Hence the efficient co-operation of hospitals and allied organisations includes large interests, and it is an aim towards which both economists and philanthropists may equally address themselves.

It may now be asked whether the same principles cannot with equal advantage be applied to the scientific work of these institutions. Medical practitioners know—and it would be well were the general public educated to the same position—that it is mainly from the hospitals that an increased knowledge of diseases and of the best methods of treating them is derived. It is here that large experience is gained and that opportunities for accumulating careful and systematic observations are afforded. Yet the question may fairly be raised whether these opportunities under existing conditions can be fully utilised. Individual physicians and individual surgeons doubtless work steadily and perseveringly and succeed in adding every now and then some substantial contribution to the sum of medical knowledge, and these gains become in various ways the common property of the profession. Again, it is true that some of the hospitals publish more or less regular reports of the work overtaken in the wards, and the statistics and comments thus accumulated are often of great value. Yet no one who is familiar with the facts will deny that large numbers of patients pass through both the in-patient and out-patient departments of the hospitals without being fully utilised as aids to the progress of the science and art of medicine. The difficulties under which the work is done are often very considerable, and many circumstances other than those of strict scientific interest demand recognition. All the same, some better organisation, and the adoption of some scheme of practical co-operation, could hardly fail at least to mitigate the existing defects. And the necessity for this is emphasised by the consideration that large opportunities mean large responsibilities, and that consequently a hospital which is failing to add regularly to the stock of medical knowledge is to this extent missing its purpose and duty.

The plea for co-operation in clinical work receives particular force when the special difficulties of the work are remembered, and when its essential position as the foundation of medical practice is recognised. Unlike the worker in the laboratory, the clinician cannot arrange and command the conditions under which his observations are conducted. He must be ready to seize an opportunity which frequently comes without warning, is often of fleeting duration, and may possibly never recur. Still more, the phenomena of disease in an individual patient are often extended over a period of months or years, so that different phases of the case come before different observers, and no one of these gains a complete picture of the whole process. Here, it is obvious, the enlargement of individual experience and the growth of sound knowledge alike demand some arrangement for co-operation between successive observers. Other instances could readily be quoted, but what has already been said is sufficient to show that the principles of co-ordination and co-operation must be recognised in the clinical and scientific aspects of hospital work if these institutions are to fulfil with success one of the great purposes of their existence.

